# [Expression of Concern] PirB restricts neuronal regeneration in developing rat brain following hypoxia-ischemia

**DOI:** 10.3892/mmr.2025.13688

**Published:** 2025-09-18

**Authors:** Hua Wang, Ying Xiong, Dezhi Mu

Mol Med Rep 6: 339–344, 2012; DOI: 10.3892/mmr.2012.907

Following the publication of this paper, it was drawn to the Editor's attention by a concerned reader that, regarding the immunofluorescence images shown in Fig. 3, the ‘HIBD’ panel appeared to share an overlapping section of data with the adjoining ‘HIBD+anti PirB’ panel, albeit the data held in common appeared to have been vertically stretched in the latter panel. The authors were contacted by the Editorial Office to offer an explanation for this apparent anomaly in the presentation of the data in this paper; however, up to this time, no response from them has been forthcoming. Owing to the fact that the Editorial Office has been made aware of potential issues surrounding the scientific integrity of this paper, we are issuing an Expression of Concern to notify readers of this potential problem while the Editorial Office continues to investigate this matter further.

